# Observation of the effect of intensity-modulated radiotherapy combined with microwave diathermy on tumor marker levels and prognosis in cervical cancer

**DOI:** 10.3389/fmed.2025.1621425

**Published:** 2025-06-25

**Authors:** Xiangdong Liu, Wei Li, Jie Zhou

**Affiliations:** ^1^Department of Oncology, Bijie Cancer Hospital, Bijie, China; ^2^Department of Integrated Traditional Chinese and Western Medicine, Bijie Cancer Hospital, Bijie, China

**Keywords:** intensity-modulated radiation therapy, microwave diathermy, tumor marker levels, prognosis, cervical cancer

## Abstract

**Objective:**

This study aimed to explore the effects of intensity-modulated radiation therapy (IMRT) combined with microwave diathermy (MWD) on tumor marker levels and prognosis in patients with cervical cancer.

**Methods:**

A retrospective analysis was conducted on the clinical data of 121 cervical cancer patients admitted to our hospital from April 2021 to April 2023. Among them, 59 patients received IMRT alone (IMRT group), while 62 patients received IMRT combined with MWD treatment (combination group). Comparison of disease control rates, serum tumor marker levels before and after treatment, quality of life (QoL), and prognosis between the two groups.

**Results:**

The combination group achieved a significantly higher disease control rate (88.71%) than the IMRT group (74.58%). After treatment, serum levels of carcinoembryonic antigen (CEA), squamous cell carcinoma antigen (SCCA), and carbohydrate antigen 50 (CA50) were significantly lower in the combination group than in the IMRT group (*p* < 0.05). Similarly, after treatment, serum levels of transforming growth factor-*β* (TGF-β), basic fibroblast growth factor (bEGF), and high-mobility protein A1 (HMGA1) were significantly reduced in the combination group than in the IMRT group (*p* < 0.05). After treatment, the improvement in the QoL in the combination group was significantly higher than that in the IMRT group (*p* < 0.05). Furthermore, the local recurrence rate (3.23%) and distant metastasis rate (1.61%) in the combination group were lower than those observed in the IMRT group (13.56% for both; *p* < 0.05).

**Conclusion:**

Compared to IMRT alone, the combination of IMRT and MWD treatment for cervical cancer patients can significantly downregulate tumor markers and levels of TGF-*β*, bEGF, and HMGA1, increase tumor control effectiveness and patient QoL, and improve disease prognosis.

## Introduction

Cervical cancer is one of the most common malignant tumors affecting women, ranking second only to breast cancer (1, 2). In recent years, the incidence of cervical cancer has increased, but early detection and prevention efforts have led to a decline in the number of advanced-stage diagnoses (3). Cervical cancer has a relatively hidden onset and lacks typical symptoms in its early stages, resulting in many patients being diagnosed in the middle or late stages, making it difficult to receive surgical treatment (4).

Intensity-modulated radiation therapy (IMRT), an advanced form of three-dimensional conformal radiotherapy, has become an important treatment measure for patients with mid-to-late-stage cervical cancer. It offers advantages such as precise positioning, accurate treatment, and enhanced safety (5). The dose intensity in the irradiation field can be adjusted according to the treatment plan, and damage to the surrounding tissues can be minimized while ensuring the radiation density of the target lesion (5, 6). According to the treatment plan, the dose intensity within the irradiation field can be precisely adjusted to maximize the radiation dose delivered to the target lesion while minimizing exposure to surrounding healthy tissues (7, 8).

In addition, the abnormal expression of transforming growth factor-*β* (TGF-β) has been shown to accelerate tumor angiogenesis, thereby affecting cervical cancer invasion and metastasis (9). Basic fibroblast growth factor (bEGF) is a common positive regulator of angiogenesis, with abnormally high expression in cervical cancer and a close correlation with microvascular proliferation (10). High-mobility protein A1 (HMGA1) can affect the transcription of tumor-related genes through different pathways, and its increased expression can accelerate tumor infiltration and metastasis, which is not conducive to disease prognosis (11). Although the markers used in this study are not specific to cervical cancer, they are widely recognized as reflective of tumor burden and treatment response across many malignancies. For example, carcinoembryonic antigen (CEA) is associated with systemic tumor activity; squamous cell carcinoma antigen (SCCA) is commonly elevated in squamous epithelial cancers, and biomarkers such as TGF-*β*, bEGF, and HMGA1 are associated with angiogenesis, immune modulation, and metastatic potential, respectively. These biomarkers serve as valuable indicators of disease progression and therapeutic response. Therefore, this study aimed to retrospectively analyze the clinical data from cervical cancer patients admitted to our hospital, with the aim of clarifying the impact of IMRT combined with MWD on the treatment efficacy and tumor marker levels.

## Materials and methods

### General information

A retrospective analysis was conducted on clinical data from 121 cervical cancer patients admitted to our hospital between April 2021 and April 2023. According to the treatment method, patients who only received IMRT will be included in the IMRT group, while patients treated with IMRT combined with MWD will be included in the combination group. Since 2020, baseline testing of tumor markers and quality of life (QoL) assessments have been part of routine clinical evaluation for newly diagnosed cervical cancer patients at our hospital. As a standard protocol, all patients underwent pre-treatment blood testing for CEA, SCCA, CA50, TGF-*β*, bEGF, and HMGA1. Baseline QoL was assessed using the European Organization for Research and Treatment of Cancer Quality of Life Questionnaire-Core 30 (EORTC QLQ-C30), which is integrated into the electronic medical record system. All patients included in this study had completed these assessments before initiating treatment.

### Inclusion criteria

Patients were eligible for inclusion if they met all of the following criteria: (1) Diagnosis of cervical cancer according to recognized clinical guidelines (1), (2) Histopathological confirmation via surgical specimen or biopsy, (3) Karnofsky Performance Status (KPS) score ≥ 70, (4) FIGO stage between IIb and IIIb, (5) Underwent treatment with IMRT alone or IMRT combined with MWD, (6) Availability of complete clinical data.

### Exclusion criteria

Patients were excluded based on the following conditions: (1) Presence of metastatic tumors, (2) Presence of other primary tumors, (3) Existence of serious underlying diseases, (4) History of alcohol and drug dependence, (5) Breastfeeding or pregnant women, and (6) History of radiation and chemotherapy treatment.

### IMRT

CT scan (layer thickness of 3 mm) was performed, with a scanning range of approximately 5 cm from the lower edge of the second lumbar vertebra to the lower edge of the obturator; the image was scanned and transferred to the post-processing system to delineate the gross tumor target area and clinical tumor target area (CTV); based on CTV, approximately 0.5 cm was expanded to create a planned target area while outlining normal tissue; using a linear accelerator electric multi-leaf grating was set (6–10 irradiation fields); once a day, 1.8 Gy/time, 5 times/week, for a total of 30 treatments, with a total dose of 54 Gy.

### MWD

Patients with MWD were selected based on high-risk tumor characteristics, including large lesion volume, poor differentiation, or elevated angiogenic activity; making the treatment decision requires a multidisciplinary tumor board discussion and patient-informed consent. MWD was performed using a local deep pelvic hyperthermia device (HG-2000 high-frequency extracorporeal hyperthermia machine), with each session lasting 60 min and conducted twice weekly; the target temperature was set at 42–43°C. MWD was administered following radiotherapy, during which the PTV dose was increased to 30 Gy in 15 fractions.

Treatment protocol and assessment standards: Post-treatment blood sampling and QoL assessments were performed uniformly 4 weeks after the completion of radiotherapy. MWD was initiated within 30 min following each IMRT session, lasting 60 min per session, twice a week, for a total of 6 to 8 sessions depending on patient tolerance. All patients received concurrent chemoradiotherapy with cisplatin or carboplatin during IMRT, followed by intracavitary brachytherapy per institutional guidelines. The disease control rate was evaluated according to the WHO RECIST criteria; QoL was measured using the EORTC QLQ-C30 tool (lower scores indicate better QoL); and cancer staging was performed using the 2018 FIGO system.

The following information was collected: (1) baseline data, including age, BMI, pathological type, FIGO staging, lesion diameter, and degree of differentiation. (2) Disease control rate: complete remission—target lesion disappearance and persistence for ≥ 4 weeks; partial remission—target lesion reduction ≥ 50% and sustained for ≥ 4 weeks; stable, the target lesion shrinks by 25 to 49% and lasts for ≥ 4 weeks; progress—target lesion enlargement>25 or new lesions appear; disease control rate = (complete remission+partial remission+stable)/total number of cases × 100%. (3) Serum tumor marker levels: Five milliliters of fasting venous blood were extracted, centrifuged (3,000 rpm, 15 min), and the supernatant was collected, and the levels of CEA, SCCA, and CA50 were measured by electrochemiluminescence. (4) Serum levels of TGF-*β*, bEGF, and HMGA1: Fasting venous blood (5 mL) were extracted, centrifuged (3,000 rpm, 15 min), the supernatant was collected, and the levels of the above indicators were determined by radioimmunoassay. (5) QoL was assessed using the EORTC QLQ-C30. The instrument includes five functioning scales, three symptom scales, a global health status scale, and several single-item measures. To facilitate a standardized comparison, we reversed the scores of all functioning items (Q1–Q7, Q20–Q24) and global health status items (Q29–Q30), making higher scores indicative of poorer QoL or greater symptom burden. A total score was calculated by summing the raw scores of all 30 items (range: 30–126), with lower scores reflecting better overall QoL. (6) Prognosis: local recurrence rate, distant metastasis rate, and 1-year survival rate.

### Statistical methods

Data were entered into Microsoft Excel and analyzed using SPSS version 26.0 (IBM Corp, Armonk, NY, USA). Normally distributed measurement data are represented as the mean ± standard deviation (SD), and *t*-tests were used to compare two independent samples between groups. For categorical variables, the frequency distribution was expressed as a percentage. The chi-squared test was used to compare categorical variables between the two groups. The differences were considered statistically significant at a *p*-value of < 0.05.

## Results

The study included 121 patients ranging in age from 43 to 78 years, with an average of 58.93 ± 7.66 years. There were 62 and 59 patients in the joint and IMRT groups, respectively. There was no statistically significant difference in baseline data between the two groups (*p* > 0.05) (Table 1).

**Table 1:** Comparison of baseline data between the two groups.

Baseline data	Combined group (*n* = 62)	IMRT group (*n* = 59)	*t*/χ^2^	*p*-value
Age (year)	58.29 ± 6.71	59.59 ± 8.55	−0.929	0.355
BMI (kg/m^2^)	23.40 ± 2.66	22.85 ± 3.05	1.068	0.288
Pathological type				
Squamous cell carcinoma	34 (54.84)	29 (49.15)	2.007	0.367
Adenocarcinoma	25 (40.32)	23 (38.98)		
Adenosquamous cell carcinoma	3 (4.84)	7 (11.87)		
FIGO staging				
IIb	31 (50.00)	31 (52.54)	2.079	0.354
IIIa	19 (30.65)	12 (20.34)		
IIIb	12 (19.35)	16 (27.12)		
Lesion diameter (cm)	3.53 ± 1.45	3.39 ± 1.38	0.554	0.580
Degree of differentiation				
Low differentiation	15 (24.19)	10 (16.95)	1.331	0.514
Middle differentiation	37 (59.68)	36 (61.02)		
Highly differentiated	10 (16.13)	13 (22.03)		

The disease control rate in the combination group (88.71%) was higher than in the IMRT group (74.58%) (*p* < 0.05) (Table 2).

**Table 2:** Comparison of disease control rates between the two groups.

Group	*n*	Complete remission	Partial remission	Stable	Progress	Disease control rate
Combined group	62	10 (16.13)	39 (62.90)	6 (9.68)	7 (11.29)	55 (88.71)
IMRT group	59	5 (8.47)	26 (44.07)	13 (22.03)	15 (25.42)	44 (74.58)
*χ* ^2^						4.059
*p*-value						0.044

**Figure 1:**
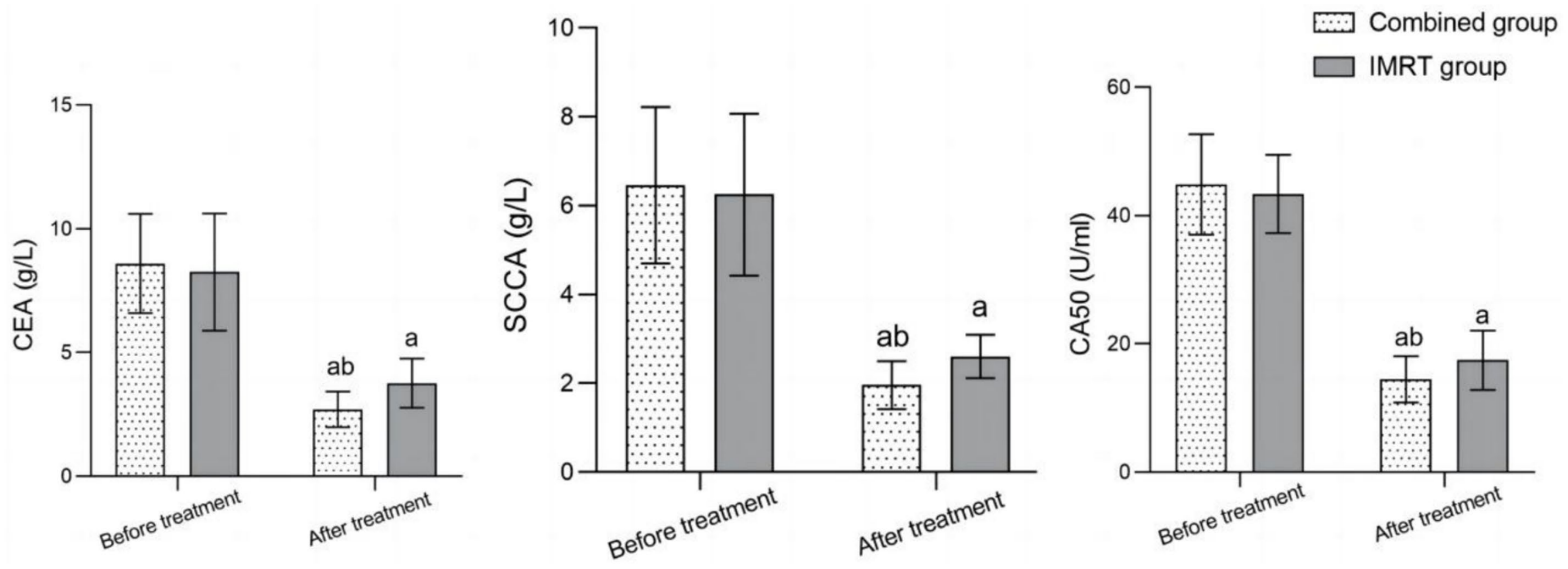
Comparison of serum tumor marker levels before and after treatment between the two groups; comparison with before treatment in the same group, ^a^*p* < 0.05; compared to the IMRT group, ^b^*p* < 0.05; IMRT, intensity-modulated radiation therapy; CEA, carcinoembryonic antigen; SCCA, squamous cell carcinoma antigen; CA50, carbohydrate antigen 50.

Before treatment, there was no significant difference in serum CEA, SCCA, and CA50 levels between the two groups (*p* > 0.05). After treatment, the serum levels of CEA, SCCA, and CA50 in both groups significantly decreased compared to before treatment, and the combined group was significantly lower than the IMRT group (*p* < 0.05) (Figure 1).

**Figure 2:**
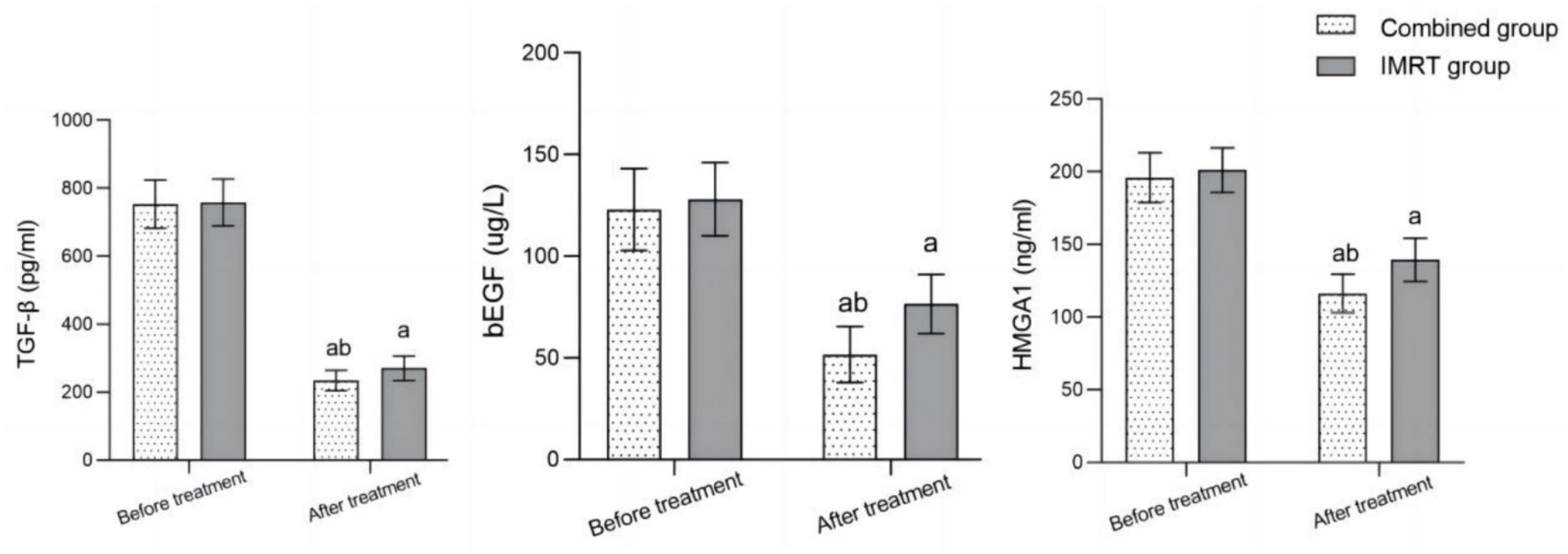
Comparison of serum TGF-β, bEGF, and HMGA1 levels before and after treatment between the two groups. Comparison with before treatment in the same group, ^a^*p* < 0.05; Compared to the IMRT group, ^b^*p* < 0.05; IMRT, intensity-modulated radiation therapy; TGF-β, transforming growth factor-β; bEGF, basic fibroblast growth factor; HMGA1, high-mobility protein A1.

Before treatment, there was no significant difference in the levels of serum TGF-*β*, bEGF, and HMGA1 between the two groups (*p* > 0.05). After treatment, the serum levels of TGF-β, bEGF, and HMGA1 in both groups significantly decreased compared to before treatment, and the combined group was significantly lower than the IMRT group (*p* < 0.05) (Figure 2).

**Figure 3:**
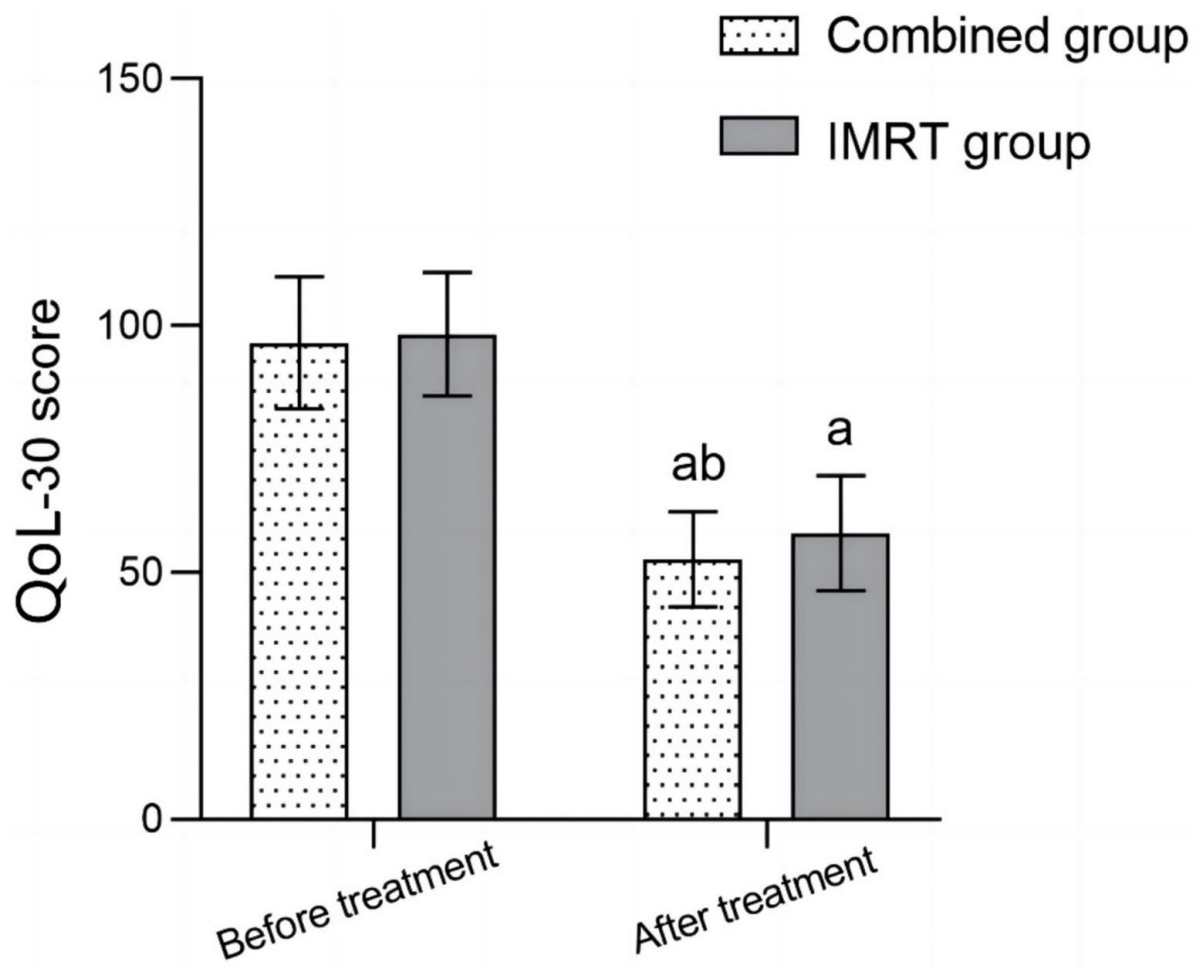
Comparison of quality of life between the two groups before and after treatment; Comparison with before treatment in the same group, ^a^*p* < 0.05; compared to the IMRT group, ^b^*p* < 0.05; IMRT, intensity-modulated radiation therapy; QoL-30, quality of life measure for oncology.

Before treatment, there was no significant difference in the QoL scores between the two groups (*p* > 0.05). After treatment, the QoL scores of the two groups significantly decreased compared to before treatment, and the combination group was significantly lower than the IMRT group (*p* < 0.05) (Figure 3).

The local recurrence rate (3.23%) and distant metastasis rate (1.61%) in the combination group were significantly lower than those in the IMRT group (13.56% and 13.56%, respectively) (*p* < 0.05). Although the 1-year survival rate was slightly higher in the combination group (96.77%) compared to the IMRT group (91.53%), the difference was not statistically significant (*p* > 0.05) (Table 3).

**Table 3:** Comparison of prognosis between two groups.

Group	*n*	Local recurrence rate	Distant metastasis rate	One-year survival rate
Combined group	62	2 (3.23)	1 (1.61)	60 (96.77)
IMRT group	59	8 (13.56)	8 (13.56)	54 (91.53)
*χ* ^2^		4.258	4.652	0.717
*p-*value		0.039	0.031	0.397

## Discussion

The results of this study showed that the disease control rate in the combination group (88.71%) was higher than that in the IMRT group (74.58%). After treatment, the serum levels of CEA, SCCA, CA50, TGF-*β*, bEGF, HMGA1, and QoL scores in both groups were lower than those in the IMRT group (*p* < 0.05). These results suggest that combining IMRT with MWD may enhance therapeutic efficacy by more effectively downregulating tumor markers and the levels of TGF-β, bEGF, and HMGA1, improving tumor control, and enhancing the QoL of patients. Follow-up studies on patients found that the local recurrence rate (3.23%) and distant metastasis rate (1.61%) in the combination group were significantly lower than those in the IMRT group (13.56 and 13.56%, respectively) (*p* < 0.05). The 1-year survival rate (96.77%) was slightly higher than in the IMRT group (91.53%) (*p* > 0.05).

Several studies have demonstrated that hyperthermia may modulate the expression of factors such as TGF-*β* and HMGA1 through effects on DNA damage repair and inflammatory signaling pathways (11, 12). Although data on cervical cancer are limited, similar findings in breast and head-and-neck squamous cancers support the suppressive effects of hyperthermia on angiogenesis and invasion markers (13, 14). This evidence supports the observed synergistic benefits of combining IMRT with MWD.

These findings further support the clinical value of combining IMRT with MWD in the treatment of cervical cancer, which helps reduce the rates of local recurrence and distant metastasis of the disease. To improve prognosis and prolong survival, MWD can help regulate the internal environment of tumor lesions, inhibit DNA repair in tumor cells, enhance their sensitivity to radiation, and have a synergistic effect with radiotherapy (15–17). Yang et al. (15) also pointed out that MWD may improve the immune function of the body by boosting the immune clearance effect of dendritic cells and macrophages on tumor lesions, upregulating the expression of heat shock proteins, and indirectly inducing apoptosis of tumor cells. A retrospective study by Jiang et al. (17) on the application value of local hyperthermia in locally advanced cervical cancer found that the objective response rates of patients reached 100.0 and 92.1% at 3 and 6 months after treatment, respectively. The local control rates at 1 year, 2 years, and 3 years after treatment were 75.85, 61.2, and 51.3%, respectively. The 1-year control rate was slightly lower than the 88.71% in this study, mainly because the statistical time of the two research results is different, and the patient’s lesion may undergo new changes, which leads to certain differences in the research results. In addition, Jiang et al. (13) also found that the 1-year survival rate after treatment reached 95.0%, which is consistent with the 96.77% in this study. Confirming the feasibility of combining MWD with conventional treatment for cervical cancer can improve the treatment efficacy and prolong patient survival (18, 19). Gao et al. (20) found that MWD can affect cell membrane permeability, structure, and function through high-temperature action, promoting the effective entry of chemotherapy drugs into tumor lesions. Moreover, hyperthermia can promote drug DNA cross-linking, enhance the killing effect of cancer cells, and enhance the sensitivity of tumor lesions to radiotherapy and chemotherapy. It was found that after combining conventional chemotherapy with the MWD treatment for advanced high-risk cervical cancer, the 5-year survival rate was only 58.0%, whereas the incidence of chronic nephrotoxicity reached 35% at the last follow-up. However, this study did not investigate the occurrence of toxic side effects in the two groups, which is also a limitation of this study. Wang et al. (21) investigated 373 cervical cancer patients, and the results showed that the 5-year survival rate of synchronous radiotherapy and chemotherapy combined with the MWD treatment increased from 72.3% for radiotherapy and chemotherapy alone to 81.9%, confirming that MWD can help improve the prognosis of cervical cancer patients. This is consistent with the results of the present study. Mei et al. (22) also confirmed that the characteristic of MWD is its sensitivity to hypoxic centers, which not only directly kills tumor cells but also has a radiosensitization effect. Moreover, the high-fever effect can inhibit the DNA double-stranded repair of tumor lesions after radiotherapy and chemotherapy, and the two modalities may exert synergistic effects when used in combination.

All serum biomarker evaluations were conducted prior to any salvage or second-line treatments. Although some patients with progressive disease subsequently received additional therapies, these treatments occurred after biomarker sampling and thus did not impact the results. This timing minimized potential bias in evaluating treatment responses based on biomarker changes.

### Limitations

First, this study was a single-center retrospective analysis that only included patients with complete clinical data, which may have led to selection bias. Second, as this was a retrospective and non-randomized study, potential selection bias cannot be fully excluded. However, strict inclusion criteria were applied, and baseline characteristics were statistically comparable between groups (see Table 1), which helps to mitigate confounding effects. Third, this study did not assess treatment-related adverse effects due to a lack of systematic toxicity documentation in the retrospective records. Future prospective studies will address both acute and chronic toxicity outcomes. In addition, the 1-year follow-up period is relatively short and insufficient to evaluate long-term outcomes such as 3- or 5-year survival, late recurrence, or metastasis. Therefore, the application value and safety of IMRT combined with MWD in cervical cancer require further clinical exploration and confirmation.

## Conclusion

Compared with IMRT alone, the combination of IMRT and MWD more effectively downregulates tumor markers and the levels of TGF-*β*, bEGF, and HMGA1 in patients with cervical cancer, improves tumor control effectiveness and patient QoL, and facilitates the improvement of disease prognosis.

## Data Availability

The original contributions presented in the study are included in the article/supplementary material, further inquiries can be directed to the corresponding author.
